# Giant cell tumour of the distal radius/ulna: response to pre-operative treatment with short-term denosumab

**DOI:** 10.1186/s13569-017-0085-3

**Published:** 2017-11-30

**Authors:** Catherine L. McCarthy, Christopher L. M. H. Gibbons, Kevin M. Bradley, A. Bass Hassan, Henk Giele, Nicholas A. Athanasou

**Affiliations:** Nuffield Orthopaedic Centre, Windmill Road, Headington, Oxford, OX3 7HE UK

**Keywords:** Giant cell tumour of bone, Distal forearm, Denosumab

## Abstract

**Background:**

Treatment of giant cell tumour of bone (GCTB) of the distal radius/ulna poses a surgical challenge, as complex reconstructive surgery may be required. This study evaluates the clinical, radiological and pathological findings in five cases of GCTB of the distal forearm where a 3 month course of denosumab was given prior to surgery.

**Methods:**

Patients with biopsy proven distal forearm GCTB, treated for 3 months with denosumab, followed by salvage surgery (curettage and cementation) were included. Wrist pain and function were assessed using the modified Mayo Wrist Score (MMWS). Plain radiographs, MRI and PET/CT were performed pre-treatment and 2 months after initiation of denosumab therapy. Histological comparison was made between the original biopsy and surgical curettage specimens.

**Results:**

Five patients with an average age of 25 years were included in the study. Improvement in wrist pain and function was seen in all patients with the average MMWS increasing from 30 pre-treatment to 85 at 3 months. Plain radiographs demonstrated marginal sclerosis in all cases with reconstitution of cortical and subarticular bone by 2 months; internal matrix sclerosis and osseous consolidation was more variable. Increased tumour heterogeneity and low signal were observed on T2-weighted MR images. PET/CT revealed a decrease in average SUV from 14.8 pre-treatment to 4.7 at 2 months. Histology showed disappearance of osteoclasts and increased fibro-osseous tissue. Denosumab treatment has the potential to facilitate salvage surgery, thus avoiding bone resection and graft reconstruction. A good outcome was achieved apart from local recurrence in one case. Follow up ranged from 17 to 54 months.

**Conclusion:**

Distal forearm GCTB responds clinically, radiologically and histologically to a short course of pre-operative denosumab therapy, which has the potential to facilitate salvage surgery.

## Background

Giant cell tumour of bone (GCTB) is a benign but locally aggressive neoplasm, accounting for approximately 5% of primary bone tumours [1–3]; it commonly develops in the mature skeleton involving the epiphyseal region of long bones. The three most common locations where GCTB develops are the distal femur, proximal tibia and distal radius respectively.

In GCTB there is a proliferation of mononuclear stromal cells which highly express receptor activator of nuclear factor kappa-B ligand (RANKL), and an infiltrate of mononuclear macrophage-like cells and scattered multinucleated osteoclastic giant cells, both of which express RANK [1, 2, 4, 5]. In the presence of macrophage-colony stimulating factor, RANK + macrophages interact with RANKL + stromal cells to induce osteoclast formation. The numerous osteoclastic giant cells in GCTB are responsible for the extensive osteolysis that attends GCTB tumour growth. Denosumab is a human monoclonal antibody that targets and binds with high affinity and specificity to RANKL; it competitively inhibits RANK–RANKL binding, decreasing the formation, activity and survival of osteoclastic giant cells in GCTB, resulting in reduced bone resorption [5–8].

Traditionally, GCTB has been treated surgically by local curettage, with or without packing of the defect with polymethylmethacrylate (PMMA) cement or bone graft, and internal fixation when needed [9–12]. The aim of this approach is to remove the tumour whilst preserving normal anatomy and wrist function. The reported recurrence rate of GCTB following this treatment is, however, relatively high with more frequent recurrence noted in the distal radius and ulna [2, 3, 12–16]. Previous studies have shown that pre-operative denosumab therapy makes subsequent surgery more feasible and may result in surgical down-staging to a less morbid surgical salvage procedure [17, 18] allowing for improved patient function. There are currently no specific guidelines on the optimal duration of pre-operative denosumab therapy and recognising the imaging features of a positive tumoral response to denosumab is important for therapeutic decision making.

This study evaluates the clinical, radiological and pathological findings in five cases of GCTB of the distal forearm that received a planned short 3 month course of denosumab prior to surgery.

## Methods

A retrospective review of patients presenting between 2012 and 2015 with GCTB of the distal upper extremity was conducted at our institution. Patients with biopsy proven (Campanacci grade 2/3) GCTB of the distal ulna or radius treated for 3 months with denosumab followed by surgical curettage and cementation, and with more than 12 months follow up, were included in the study. Excluded patients were those that lacked a pre-treatment biopsy and/or had received prior treatment for their GCTB, including denosumab treatment for more than 3 months, and/or had > 12 months follow-up.

Denosumab was administered by subcutaneous injection of 120 mg every 4 weeks with additional loading doses of 120 mg on days 8 and 15 of the first month. Daily supplements of calcium 500 mg and vitamin D 400 IU were taken and serum electrolytes were checked every 4 weeks. This was performed for a total of three cycles.

Clinical data collected included age, sex, location of tumour, length of follow-up and complications post-surgery. Patient wrist pain and function were assessed by the medical oncologist administering denosumab at the commencement of treatment and at 4 weekly intervals for the 3 month treatment using the modified Mayo Wrist Score (MMWS). Each assessment totaled 100 points divided among wrist pain (25 points), range of motion as a percentage of the opposite side (25 points), grip strength as a percentage of the opposite side (25 points), and the ability to return to regular employment or activities (25 points).

All patients were initially assessed with plain radiographs, magnetic resonance imaging, with and without gadolinium contrast (MRI), and positron emission tomography with fluorodeoxyglucose integrated with computed tomography (PET/CT). These studies were repeated 2 months after the commencement of denosumab therapy. All patients had post-operative and follow-up plain radiographs. All imaging studies were repeated if there was clinical or radiographic suspicion of recurrence.

Following 3 months denosumab treatment, surgical management consisted of careful and thorough curettage of all clinically visible disease, including bony septa and the neo-sclerotic intra-osseous margin, then painting the cavity with methylene blue and high speed burring of the sclerotic internal margins of the tumour. PMMA cement was used to fill the small defects created in the bone. No other adjuvants such as phenol or cryotherapy were used. The same sarcoma surgeon, with over 30 years experience, treated and followed up all cases.

In assessing the histological response to denosumab, comparison was made between the original biopsy specimen and surgical curettage specimens. The presence or absence of osteoclasts, the number of mononuclear stromal cells and the extent of fibrous matrix/bone formation was noted.

## Results

Between May 2012 and July 2015, five patients fulfilling the inclusion criteria were referred to our unit with GCTB of the distal forearm. There were four females and one male with an average age of 25 years.

### Pre-denosumab treatment findings

#### Clinical

The patients presented with increasing wrist pain (n = 5), wrist swelling (n = 4), reduced wrist grip (n = 3), reduced wrist movement and function (n = 5). The average MMWS score prior to treatment was 30. Table 1 presents demographic, clinical and radiological data on the cases in this study.

**Table 1:** Demographic, clinical and radiological patient data

Patient #	Age	Sex	Site	Campanacci grade	Marginal sclerosis after denosumab	Intralesional matrix sclerosis after denosumab	Pre/post SUV on PET/CT	Time between denosumab and surgery (days)	Follow-up (months)	Local recurrence (time in months)
1	19	Male	Right radius	2	Yes	Moderate	19.5/8.8	23	47	No
2	21	Female	Right radius	2	Yes	Mild	7.2/3.2	18	17	No
3	39	Female	Right ulna	2	Yes	Mild	10.6/2.0	36	54	No
4	27	Female	Left radius	2	Yes	Moderate-marked	17/4.8	25	35	No
5	22	Female	Right radius	3	Yes	Moderate-marked	19.5/4.5	39	32	Yes (2)

#### Radiological

**Fig. 1:**
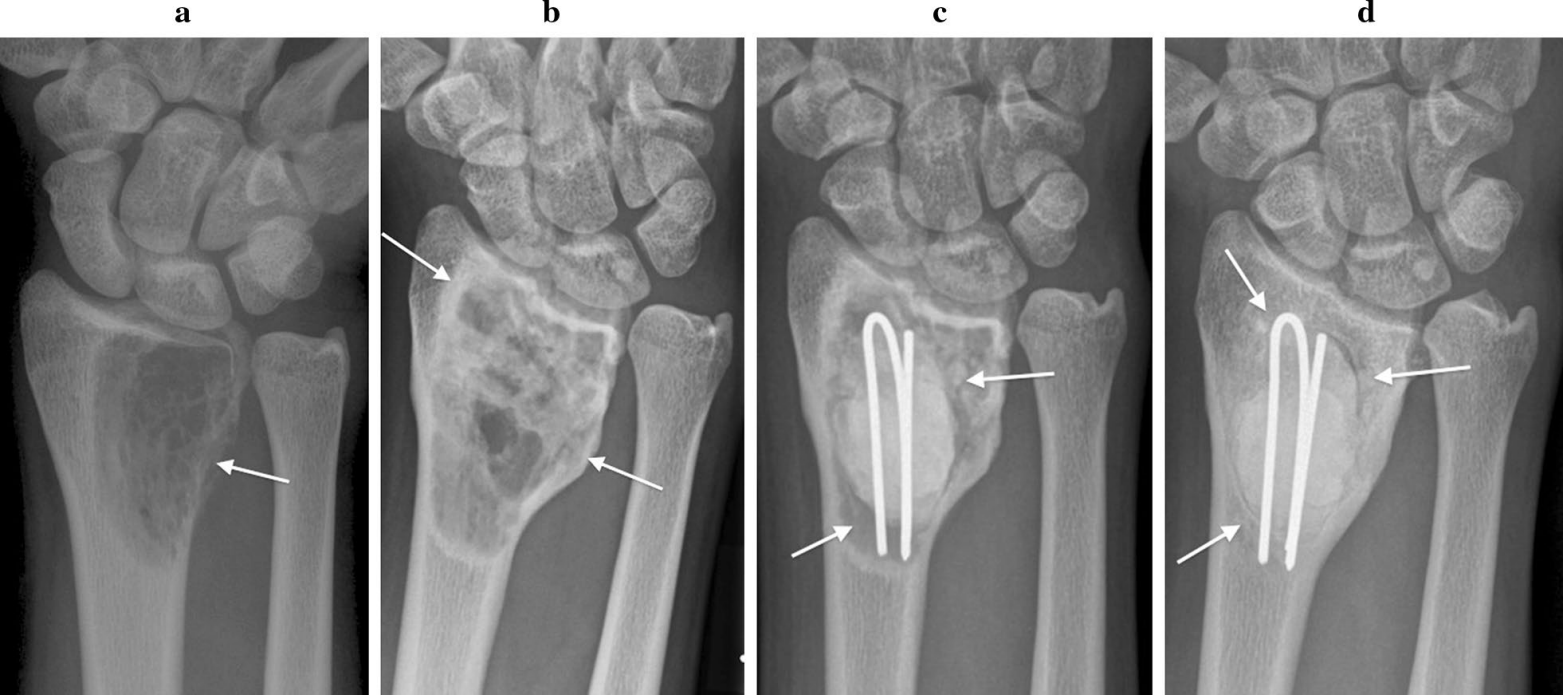
Patient 1: pre denosumab, **a** frontal plain radiograph shows a lytic Campanacci grade 2 distal radial GCTB with mild expansion and cortical thinning (arrow). Post denosumab at 2 months, **b** frontal plain radiograph demonstrates sclerosis of the tumour margin (arrows) and radial articular surface. There is mild modeling deformity of the radial articular surface and moderate internal matrix osteosclerosis. Post surgical, **c** frontal plain radiograph 1 week post surgical curettage, cementoplasty and internal fixation shows lucency of the peripheral tumour cavity (arrows) and **d** 2 years post surgery with progressive infilling of the lesion with osteosclerosis around the cement despite no post operative denosumab therapy (arrows)

**Fig. 2:**
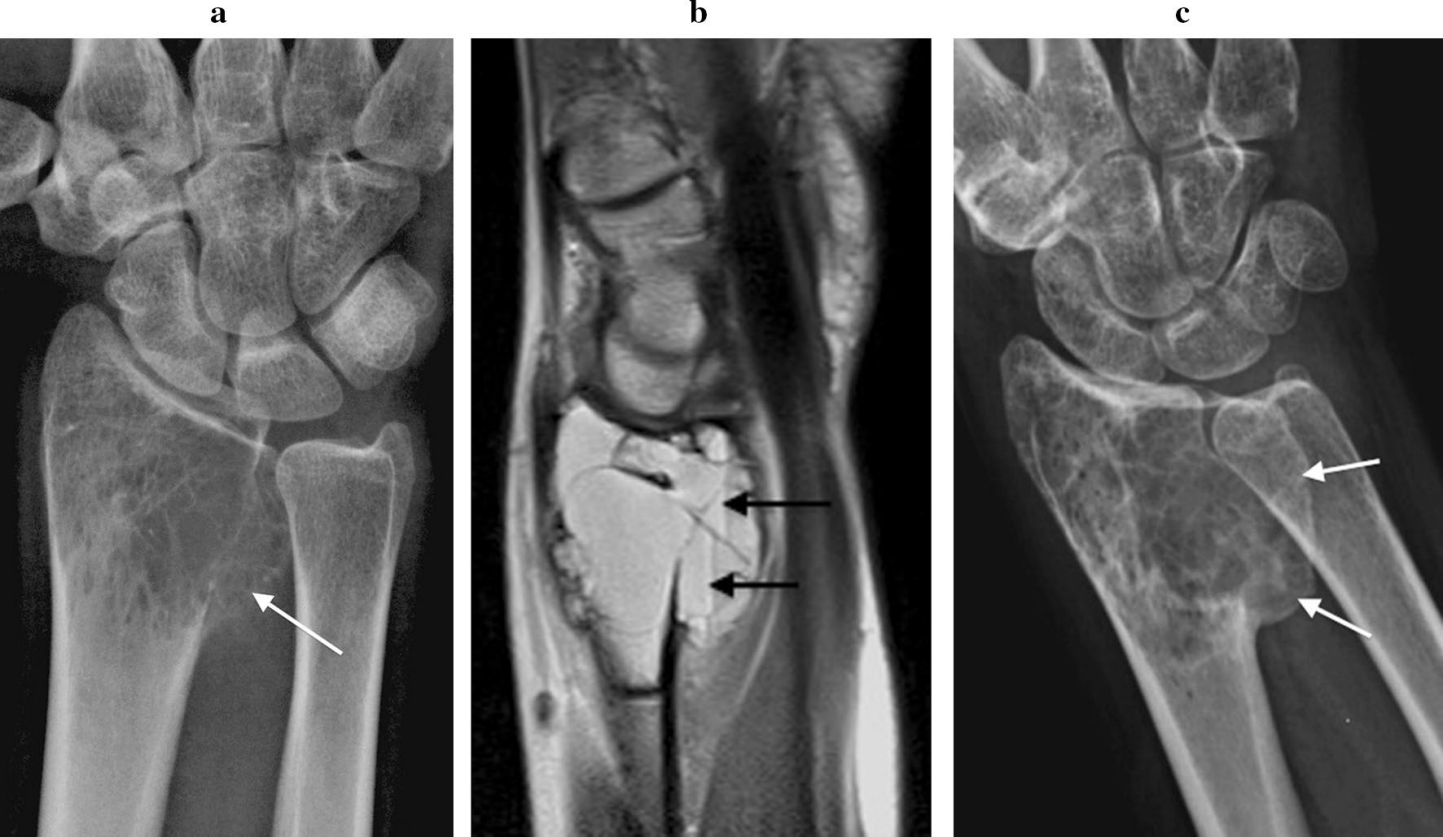
Patient 2: pre denosumab, **a** frontal plain radiograph shows a lytic Campanacci grade 2 distal radial GCTB with medial expansion and cortical thinning (arrow). **b** Sagittal T2-weighted MR image demonstrates volar expansion and fluid fluid levels (arrows) indicating secondary aneurysmal bone cyst. Post denosumab at 2 months, **c** frontal plain radiograph demonstrates more clearly defined sclerotic tumour margins particularly along the expansile medial aspect of the lesion (arrows) and mild matrix osteosclerosis

**Fig. 3:**
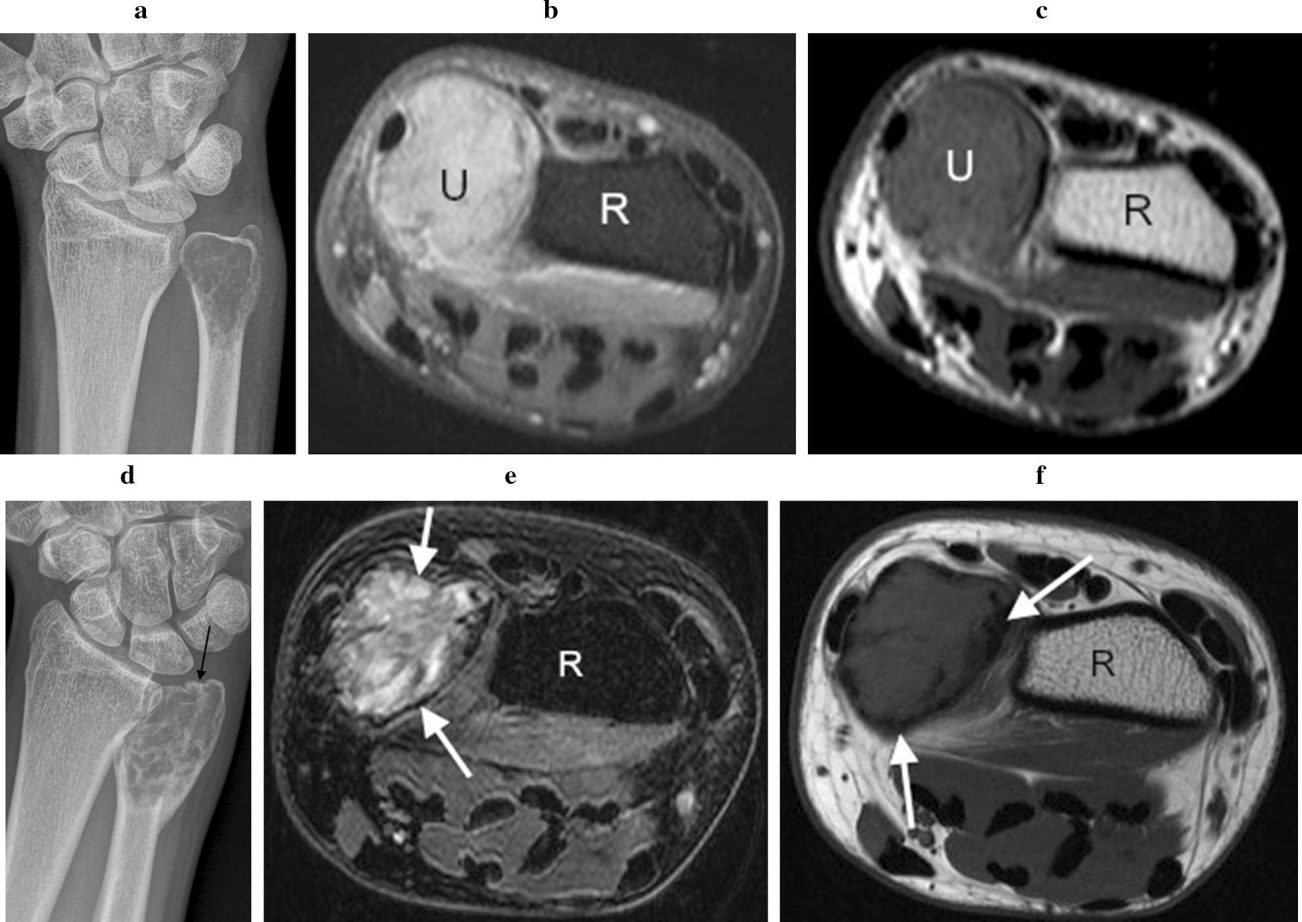
Patient 3: pre denosumab, **a** frontal plain radiograph demonstrates a Campanacci grade 2 distal ulnar GCTB with mild expansion and cortical thinning. **b** Axial T2-weighted fat saturated and **c** axial T1-weighted MR images confirm an expansile heterogeneous predominantly high T2- and low to isointense T1-weighted distal ulnar lesion (*U* ulnar, *R* radius). Post denosumab at 2 months, **d** frontal plain radiograph shows marginal sclerosis and internal matrix sclerosis, with mild modeling deformity of the ulnar articular surface (black arrow). **e** Axial T2-weighted fat saturated and **f** axial T1-weighted MR images show definition of cortical margins with the formation of marginal sclerosis seen as peripheral low T1 and T2-W signal (white arrows). The tumour matrix is more heterogeneous with areas of low T2-W signal reflecting internal matrix consolidation (*R* radius)

**Fig. 4:**
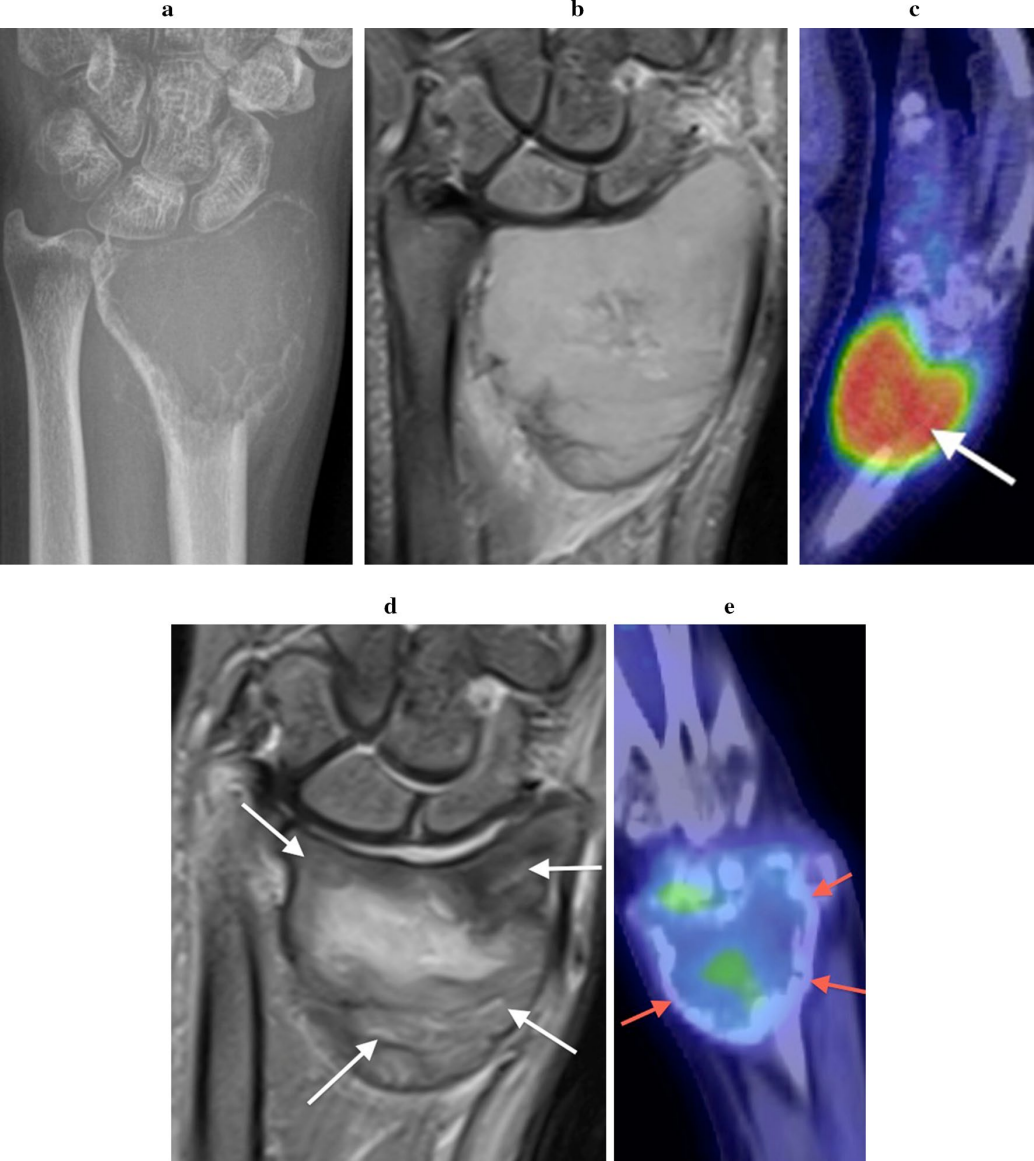
Patient 4: pre denosumab, **a** frontal plain radiograph demonstrates a lytic expansile Campanacci grade 2 distal radial GCTB with cortical thinning. **b** Coronal STIR MR image shows the lesion returns heterogeneous predominantly high STIR MR signal. **c** Fused PET/CT image illustrates marked FDG uptake with a standardized uptake value (SUV) of 17 (arrow). Post denosumab at 2 months, **d** coronal STIR MR image shows increased heterogeneity of MR signal with areas of low STIR signal reflecting matrix osteosclerosis (white arrows). **e** Fused PET/CT image confirms a significant decrease in FDG uptake with an SUV of 4.8 and the formation of marginal sclerosis (red arrows)

**Fig. 5:**
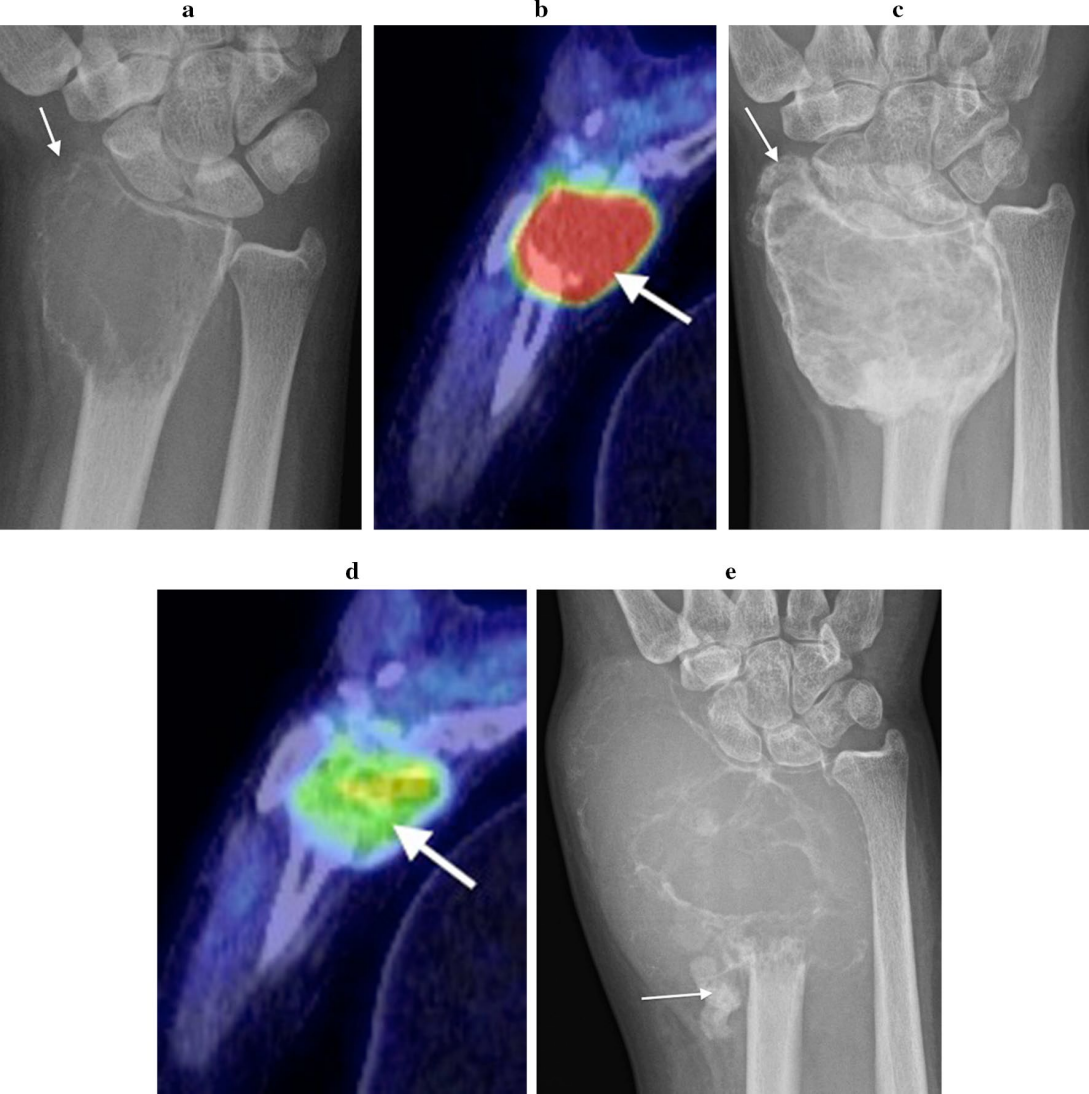
Patient 5: pre denosumab, **a** frontal plain radiograph illustrates a distal radial GCTB with moderate expansion and cortical thinning. A breach of the radial styloid articular surface (arrow) with soft tissue extending into the carpal joint was confirmed on MR imaging and this lesion was graded as Campanacci grade 3. **b** Fused PET/CT image confirms marked metabolic activity with an increased SUV of 19.5 (arrow). Post denosumab at 2 months, **c** frontal plain radiograph demonstrates an increase in size of the lesion despite marginal sclerosis with reconstitution of the radial articular surface (arrow) and quite marked intralesional matrix consolidation. **d** Fused PET/CT image confirms reduced activity of the lesion with a significant decrease in the SUV to 4.5 (arrow). Post surgical, **e** frontal plain radiograph 2 months post surgery shows tumour recurrence with rapid growth, progressive bone destruction and extrusion of cement (arrow)

Initial plain radiographs demonstrated in all cases a solitary, expansile, lytic lesion involving the epiphysis and extending up to the articular surface of the distal radius (n = 4) or ulna (n = 1). The physis was fused in all patients. Four lesions were graded as Campanacci grade 2 (active). These showed eccentric expansion of bone with associated cortical thinning but no cortical de-struction or pathologic fracture (Figs. 1a, 2a, 3a, 4a). One lesion was graded as Campanacci grade 3 (aggressive) with cortical breach of the radial styloid articular surface (Fig. 5a). MRI showed expansile subarticular lesions returning low to isointense signal relative to surrounding muscle on T1-weighted imaging (Fig. 3c) and heterogeneous predominantly high STIR and T2-weighted signal intensity (Figs. 3b, 4b). Gadolinium was administered in three cases and demonstrated diffuse enhancement in all cases. There were fluid–fluid levels in one patient indicative of secondary aneurysmal bone cyst change (Fig. 2b). There was a focal breach of the radial styloid articular surface with a small amount of soft-tissue extending into the radial aspect of the carpal joint in the Campanacci grade 3 lesion. In the remaining cases, the articular surface appeared intact. PET/CT revealed lytic, expansile lesions without a sclerotic rim and marked cortical thinning with an increased standardized uptake value (SUV) in all cases (Figs. 4c, 5b). The average pre-treatment SUV was 14.8 (Table 1). There were no distant lesions and the lungs were clear in all patients.

#### Histological

**Fig. 6:**
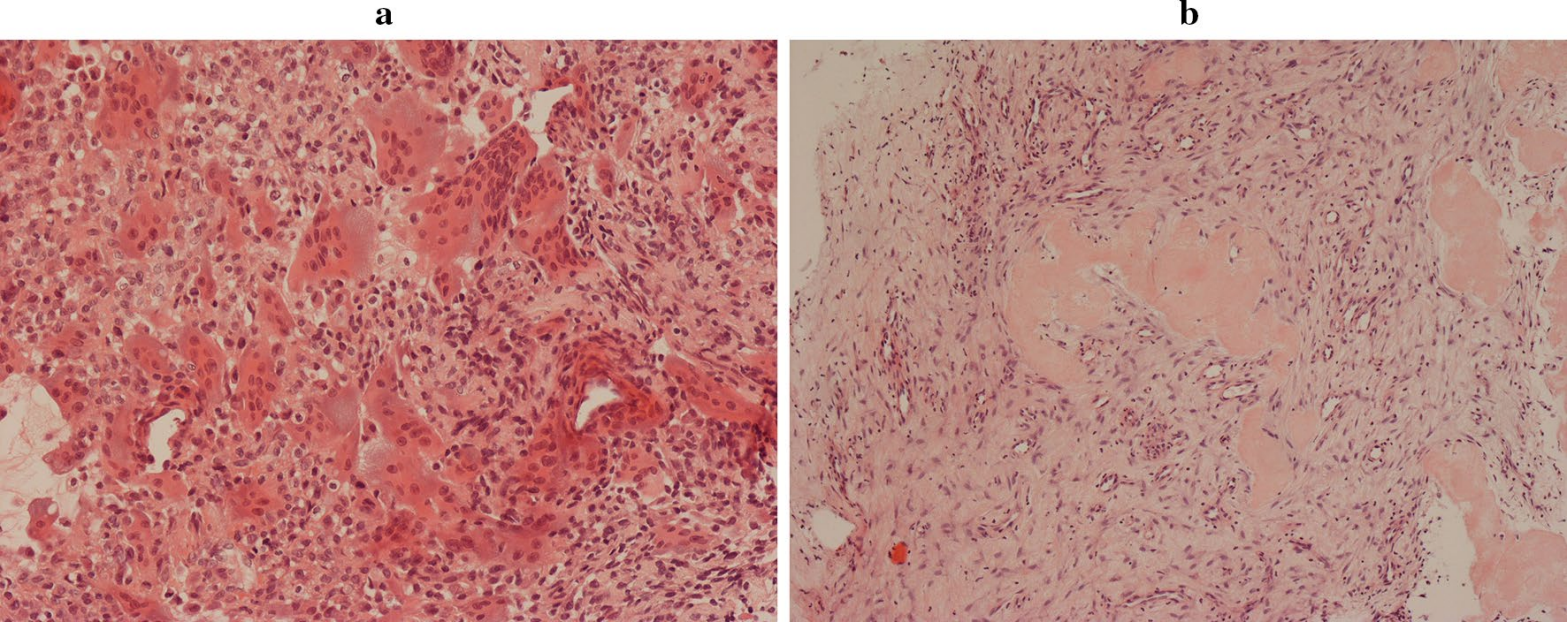
Histology: pre denosumab, **a** numerous osteoclast-like giant cells, some of which have a very large number of nuclei, and surrounding mononuclear cells. Post denosumab, **b** well-vascularised fibrous tissue in which there is focal osteoid/woven bone formation and absence of osteoclasts

The diagnosis of GCTB was histologically confirmed pre-treatment in all cases. Histology showed a proliferation of mononuclear stromal cells, numerous macrophages and frequent scattered osteoclast-like giant cells, some of which were very large and contained a large number of nuclei (Fig. 6a).

### Post-denosumab treatment findings

#### Clinical

Denosumab treatment was administrated for 3 months pre-surgery; it was generally well tolerated and the patients had no serious treatment-related adverse events. In all patients a decrease in wrist pain and improved wrist function was noted within 2 months of commencing denosumab therapy, with the average MMWS increasing to 75. The MMWS increased further to 85 at 3 months.

#### Radiological

Two months following the commencement of denosumab treatment, plain radiographs showed well-defined marginal sclerosis with reconstitution and mineralization of cortical bone in all cases. Varying degrees of internal matrix sclerosis and osseous consolidation resulted in increased radiopacity of the lesions (Figs. 1b, 2c, 3d, 5c). Cortical sclerosis resulted in mild cortical irregularity and modeling deformity involving the articular surface in two cases (Figs. 1b, 3d). The Campanacci grade 2 lesions showed no significant change in tumour size. The Campanacci grade 3 lesion showed a 15% increase in tumour volume in addition to considerable marginal and matrix sclerosis after 2 months of denosumab treatment (Fig. 5c). MRI showed increased low signal marginal sclerosis with clearer definition of cortical margins (Fig. 3e, f). The articular surface appeared intact in all cases with no soft tissue mass. There was no significant change in T1 weighted signal (Fig. 3f). T2 weighted signal appeared more heterogeneous post-treatment with new areas of internal low T2 signal along with multiple central non-enhancing areas post-gadolinium (Figs. 3e, 4d). PET/CT confirmed radiologic evidence of bone repair with marginal and intralesional osteosclerosis in all patients. The average SUV at 2 months after commencing denosumab decreased to 4.7 (Figs. 4e, 5d; Table 1).

#### Histological

Histopathological examination of the treated GCTBs showed an absence of osteoclasts and an increase in well-vascularized cellular fibrous and fibrosseous tissue with focal areas of osteoid and woven bone formation (Fig. 6b); these were of variable thickness and shape and covered partly by bone lining cells. There were scattered macrophage-like cells but no giant cells were observed. There was no mitotic activity or nuclear pleomorphism of stromal cells, which appeared reduced in number within cellular fibrous tissue. The lesions were surrounded by organised reactive bone.

#### Outcome following denosumab treatment and salvage surgery

Curettage of the GCTB with insertion of PMMA cement was performed an average of 28 days after cessation of denosumab treatment. Internal fixation was used in one case due to bone destruction that necessitated support of the distal radial joint surface (Fig. 1c). There were no intraoperative complications.

Average follow-up was 37 months (Table 1). Four patients had a good clinical and functional outcome with no post-surgery complications or evidence of recurrence. One of these patients demonstrated progressive infilling of the tumour with osteosclerosis around the cement on plain radiographs over 2 years (Fig. 1d) despite no further denosumab therapy; this did not result in increased symptoms. One patient with the Campanacci grade 3 lesion had local tumor recurrence 2 months post-surgery; this was evident from radiographic imaging which showed progressive bone destruction with involvement of the radio-carpal joint (Fig. 5e). Resection of the distal radius with a vascularized free fibular graft (VFFG) and joint reconstruction was performed 7 months after initial surgery in this case. Twenty-three months after this intervention there has been no evidence of recurrence.

None of the patients has shown evidence of distal disease.

## Discussion

GCTB is a benign but locally aggressive tumour that occurs most commonly in a relatively young patient population. The distal radius is the third most common location where GCTB develops and, as in other sites, curettage of the tumour and cementation is often employed as first-line treatment [12, 16, 19–21]. However, distal radius/ulna GCTBs frequently recur following this treatment, usually within the first 2 years [22] and complex reconstructive surgery is often required, not only to limit local recurrence but also to preserve functional anatomy [12, 23].

There are a few previous case reports describing the effect of denosumab treatment on GCTB of the distal radius. A beneficial effect of denosumab was noted on a GCTB of the distal radius [24] and a locally aggressive giant cell-containing lesion of the radius diagnosed as aneurysmal bone cyst, a lesion which is also known to contain stromal cells that express RANKL [25, 26]. Matcuk et al. [27] noted rapid recurrence and aggressive growth of a GCTB after the cessation of long-term denosumab therapy, indicating that surgery is important in managing these tumours. This is the first case series examining the effect of a pre-operative short course of denosumab treatment on GCTB in bones of the distal forearm. As such, there are no guidelines on patient selection or optimal treatment in terms of dose and duration of denosumab administration; our denosumab schedule was based on previous phase 2 studies [6, 18, 28]. Denosumab was well tolerated in our cases, none of whom reported any of the known side effects of this drug [7, 8, 18].

The origin of pain in GCTB is multifactorial including mechanical stress from tumour related pressure, tumour growth with expansion of the periosteum, the loss of structurally significant bone and mechanical failure. The production of prostaglandins, endothelins and other noxious factors by the tumour cells are also known to result in pain [28]. Clinically, all five patients showed a significant decrease in wrist pain within 8 weeks of commencing denosumab therapy. This is in keeping with previous studies, which reported a clinically relevant decrease in pain with low or no analgesic use in most patients within 2 months of commencing denosumab therapy [6, 18, 28]. Improved wrist mobility and function was also noted in all patients within 8 weeks of denosumab therapy as seen in prior studies [6, 28].

Radiological features indicative of a positive response to denosumab are marginal sclerosis of the tumour and cortical thickening which was clearly seen all patients after 2 months of denosumab treatment. Similar radiological findings have been described in other studies where denosumab treatment ranged from 2 months to 2 years [27, 29–33]. One of our cases demonstrated reconstitution of cortical bone with repair of a focal defect in the articular surface. Intralesional consolidation was more variable with mild to more marked matrix osteosclerosis. In our cases, marginal and intralesional osteosclerosis was best appreciated on plain radiographs and the CT component of the PET/CT. PET/CT also revealed a decrease in average SUV from 14.8 pre-treatment to 4.7 at 2 months, indicating that PET/CT may be a sensitive monitor for the response to denosumab. MRI did not aid in evaluating the efficacy of denosumab therapy, although increased tumour heterogeneity on T2-weighted images with areas of decreased gadolinium uptake were observed. MRI findings of increased intralesional heterogeneity, cortical thickening and irregularity and linear perilesional fluid like signal following short term denosumab therapy have previously been described and misinterpreted as disease progression [33]. These MR features were present in our cases and represent an appropriate response to denosumab treatment. It has been suggested that increased MRI tumour heterogeneity and T2 low signal may reflect central necrosis and osteosclerosis in the tumour [29, 30]. Histopathological findings in our denosumab treated cases did not show evidence of necrosis but did show prominent intralesional ossification and fibrosis, disappearance of osteoclastic giant cells and a reduction in the number of mononuclear stromal cells, as previously described [6, 29, 30, 34, 35].

In one of our cases, ongoing post operative infilling and sclerosis was noted around the cement on plain radiographs despite no post-operative denosumab treatment. This case continues to have an excellent clinical and radiological response with no evidence of recurrent symptoms or tumour 3 years and 9 months post surgery. The findings in this case suggest that there may be some continued response to short term denosumab therapy.

An overall reduction in GCTB size has been noted following denosumab treatment for an average duration of 15.3 (12.1–23.6) months [17]. In our series none of the lesions decreased in size when imaged after 2 months of denosumab treatment and the Campanacci grade 3 lesion was larger than on presentation radiographs.

Local recurrence occurred in one case, the Campanacci grade 3 lesion that had aggressive radiological features pre-denosumab with involvement of the cortex, breach of the radial styloid articular surface and a small soft tissue mass evident on MRI. In this case, after 2 months of denosumab treatment, despite an overall increase in tumour size, disappearance of the soft tissue component and reconstitution of the articular cortex, was noted, as well as matrix osteosclerosis and reduced metabolic activity on PET (SUV 19.5–4.5). This allowed the patient the opportunity of initial surgical curettage with the chance for an improved functional outcome.

Previous studies have shown that denosumab results in a decrease in tumour progression, with surgical down-staging to a less morbid surgical salvage procedure than originally planned in 38% (n = 84/222; average duration of denosumab treatment 15.3 months) and 61% (n = 16/26; average duration of denosumab treatment 6 months) of patients [17, 18]. In our experience of treating GCTBs with neoadjuvant denosumab, we found that treatment for more than 3 months often resulted in marked intralesional sclerosis and extensive perilesional new bone formation. This makes curettage of some GCTBs more difficult, and, as noted by some observers [36], can create uncertainty with regard to the adequacy of tumour excision. In this study we show that a short course of denosumab results in reconstitution of cortical and subarticular bone and sufficient marginal sclerosis to define the tumour margin whilst not causing marked intralesional sclerosis. The sarcoma surgeon who treated all cases, was of the opinion that this treatment regime facilitated curettage of the lesion with preservation of the native joint, local functional anatomy and wrist function.

It has been suggested that, although the newly formed bone at the tumour periphery allows for a sufficient mechanical scaffold for curettage to be done without fear of the bone collapsing, this rim of sclerotic bone may contain neoplastic stromal cells that may become active and express RANKL after denosumab treatment is stopped [36]. Another advantage of performing curettage earlier following a short course of denosumab is prevention of too thick a rim of peripheral bone from being formed; this decreases the chances of neoplastic tissue being left behind post-curettage in the thickened rim of perilesional new bone thus facilitating complete tumour removal. A recent study of 35 patients with locally advanced Campanacci grade 3 GCTB supports a short course (3 months) of denosumab treatment prior to surgical curettage; however, postulates that a longer duration of denosumab therapy should be administered for en bloc tumour resection, which may be facilitated by more marked tumour sclerosis with a lower risk of recurrence [35].

## Conclusion

Our results indicate that GCTB of the distal forearm responds clinically, radiologically and histologically within 3 months of denosumab treatment. Short term denosumab was well tolerated and thought to facilitate surgical curettage in all cases, with preservation of the native joint and a good surgical outcome apart from one case of recurrence in a Campanacci grade 3 lesion. Limitations of this study include its retrospective nature, and clearly small sample size. However, the rarity of GCTB, especially with regard to a particular anatomic site makes the reporting of larger numbers challenging in a single institution. Co-ordinated, multi-institutional studies are likely to be needed in order to fully assess this treatment regime and whether a short course of neoadjuvant denosumab would be similarly effective for GCTBs in other bones.

## Abbreviations

GCTB: giant cell tumour of bone
RANKL: receptor activator of nuclear factor kappa-B ligand
PMMA: polymethylmethacrylate
MMWS: modified Mayo Wrist Score (MMWS)
MRI: magnetic resonance imaging
PET/CT: positron emission tomography with fluorodeoxyglucose integrated with computed tomography
STIR: short tau inversion recovery
SUV: standardized uptake value
VFFG: vascularized free fibular graft
